# Label-free Raman hyperspectral imaging analysis localizes the cyanogenic glucoside dhurrin to the cytoplasm in sorghum cells

**DOI:** 10.1038/s41598-018-20928-7

**Published:** 2018-02-09

**Authors:** Philip Heraud, Max F. Cowan, Katarzyna Maria Marzec, Birger Lindberg Møller, Cecilia K. Blomstedt, Ros Gleadow

**Affiliations:** 1 Department of Microbiology, Faculty of Medicine, Nursing and Health Sciences, Monash University, Wellington Rd, Clayton, Vic., 3800 Australia; 2 Centre for Biospectroscopy, School of Chemistry, Monash University, Wellington Rd, Clayton, Vic., 3800 Australia; 3 School of Biological Sciences, Faculty of Science, Monash University, Wellington Rd, Clayton, Vic., 3800 Australia; 4 Jagiellonian Centre for Experimental Therapeutics (JCET), Jagiellonian University, Bobrzynskiego 14, Krakow, Poland; 5 Center for Medical Genomics (OMICRON), Jagiellonian University, Kopernika 7C, 31–034 Krakow, Poland; 6 Centre for Synthetic Biology, University of Copenhagen, Thorvaldsensvej 40, 1871 Frederiksberg C, Denmark; 7 VILLUM Center for Plant Plasticity, University of Copenhagen, Thorvaldsensvej 40, 1871 Frederiksberg C, Denmark

## Abstract

Localisation of metabolites in sorghum coleoptiles using Raman hyperspectral imaging analysis was compared in wild type plants and mutants that lack cyanogenic glucosides. This novel method allows high spatial resolution *in situ* localization by detecting functional groups associated with cyanogenic glucosides using vibrational spectroscopy. Raman hyperspectral imaging revealed that dhurrin was found mainly surrounding epidermal, cortical and vascular tissue, with the greatest amount in cortical tissue. Numerous “hotspots” demonstrated dhurrin to be located within both cell walls and cytoplasm adpressed towards the plasmamembrane and not in the vacuole as previously reported. The high concentration of dhurrin in the outer cortical and epidermal cell layers is consistent with its role in defence against herbivory. This demonstrates the ability of Raman hyperspectral imaging to locate cyanogenic glucosides in intact tissues, avoiding possible perturbations and imprecision that may accompany methods that rely on bulk tissue extraction methods, such as protoplast isolation.

## Introduction

Leaf composition in the past has typically been analysed by homogenizing whole leaves, or parts of leaves, removing the ability to distinguish between relative changes in composition of the different cell types present and rely on the assumption that the leaves are uniform in the distribution of chemicals across the leaf blade (e.g.^1–3^). However, plant constituents are typically distributed differently within leaves, and within or between species, such that plant plasticity and performance are enhanced^4,5^. Herbivores, for example, may eat specific parts of leaves, not entire leaves^6^. Localisation of specific metabolites can be determined microscopically using stains that identify particular functional groups, or by isolating different tissues or cell types for chemical analysis, e.g. using laser dissection^7^ or protoplast isolations^8^.

New methods for “tissue fingerprinting” are being developed e.g. based on Fourier-transform infrared microspectroscopy or mass spectrometry based bioimaging^3,6,9,10^. While powerful, these methods are dependent on fixing and preserving the plant materials. Raman microspectroscopy is a new technique that offers chemical information on the constituents present at high spatial resolution with microscopic samples analysed directly *in situ* without the need for fixing and prior chemical treatments^11,12^. It has been applied in studies to detect a range of analytes in biological tissues^13–15^.

An important feature of Raman spectroscopy is that is it able to detect nitrile groups characteristic of cyanogenic glucosides^16^. Cyanogenic glucosides are an important group of specialized metabolites that are hydrolysed with concomitant release of toxic hydrogen cyanide when mixed with specific β-glucosidases. This property renders cyanogenic glucosides effective as defence compounds against generalist herbivores^17^. In the intact plant, cyanogenesis is prevented by spatial separation at the organelle or tissue level of cyanogenic glucosides and the specific β-glucosidase enzymes required for their hydrolysis with the actual arrangement varying between species^18^. Accordingly, disruption of this spatial separation e.g. by a chewing insect results in release of toxic hydrogen cyanide^18^. The C≡N triple bond in cyanogenic glucosides gives rise to characteristic Raman spectra, with a band at approximately 2240–2250 cm^−1^ originating from the stretching vibration of the ν(C≡N) mode, with the exact wavelength depending on the specific cyanogenic glucoside^16^.

In this study, our aim was to investigate whether we could determine the *in situ* localization of dhurrin in developing sorghum tissue and image its concentration and distribution. We mapped the tissue using a Raman spectrometer and generated Raman hyperspectral images^19^, based on spatial position and spectral information related to dhurrin. To confirm the efficacy of the method, we compared Raman hyperspectral images showing the relative concentration and distribution of dhurrin in sorghum plants with normal dhurrin production (*Sorghum bicolor* L. Moench), with sorghum mutants that do not produce dhurrin^20^. These totally cyanide deficient (*tcd1*) mutants have a mutation that results in a non-functional form of the cytochrome P450 enzyme CYP79A1 that catalyses the first step of the biosynthetic pathway and offers an ideal negative control. Micklander, *et al*.^21^ used Raman spectroscopy to compare the concentration of the cyanogenic glucoside, amygdalin, in ground endosperm of bitter and sweet almonds (*Prunus amygdalus*). Raman has also been used to map the distribution of amygdalin in apricot kernels (*Prunus armeniaca*) but relied on an embedding technique^22^. Early studies to determine the localization of dhurrin in sorghum in the 1970s isolated protoplasts from the leaves and separated epidermal and mesophyll cells using density gradient centrifugation^8,23^. These studies determined that dhurrin was almost exclusively found in the vacuole of the epidermal cells of the leaf blade. Here, we combine Raman microspectroscopy with multivariate image analysis creating Raman hyperspectral imaging to visualize the concentration and distribution of cyanogenic glucosides *in planta* at sub-cellular spatial resolution, without the need for stains or chemical fixatives.

## Results and Discussion

### Raman spectroscopy of pure dhurrin

**Figure 1:**
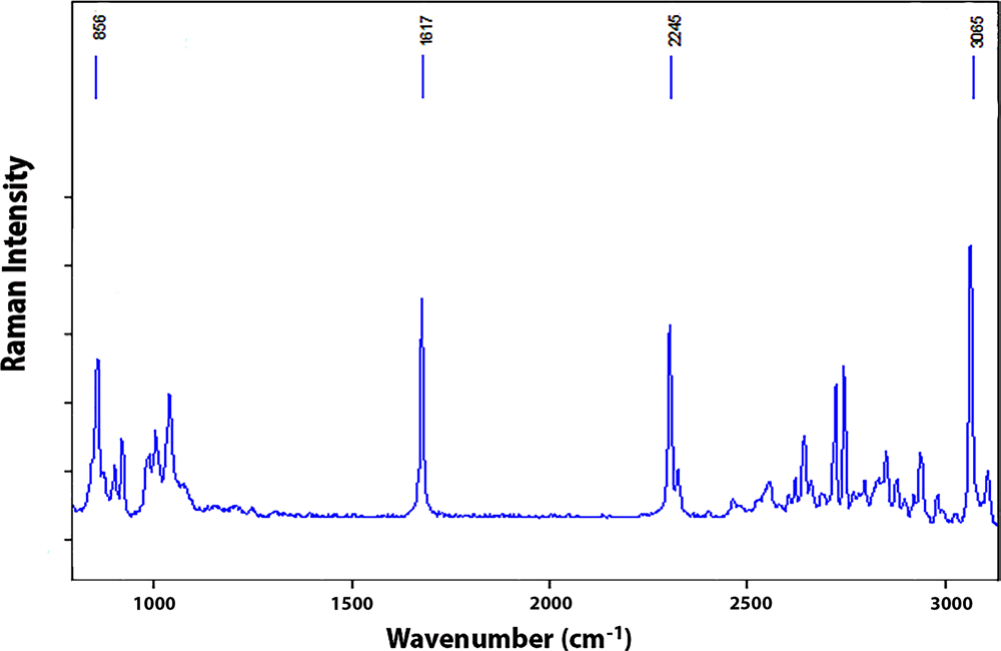
Raman spectrum of dhurrin using 532 nm excitation with 0.5 mW of laser power for 2 sec. The major bands attributed to dhurrin and their functional group vibrations and band positions (in cm^−1^) are highlighted (see Table 1 for explanation of how bands are assigned).

We measured the Raman spectrum of chemically synthesised dhurrin^24^ to be able to recognize and confirm spectral features characteristic of biogenic dhurrin in spectra from plant tissue hyperspectral image data. The spectrum of dhurrin (Fig. 1) has a band profile similar to other published Raman spectra of cyanogenic glucosides, such as amygdalin^21,22^. Prominent and common to dhurrin and all other cyanogenic glucosides is the band at 2245 cm^−1^ from the nitrile moiety (ν C≡N; Table 1). This band constitutes an unmistakable marker of cyanogenic glucosides, as the band occurs in an otherwise “quiet” region of the biological spectrum that is not populated by other bands of biological origins. Other prominent bands in the dhurrin spectrum included those at 3065 cm^−1^ and 856 cm^−1^ from out of plane stretching and bending modes of aromatic C-H groups, respectively, and the prominent aromatic C-C stretching mode at 1617 cm^−1^. Although not unique to cyanogenic glucosides and found in the spectrum of other cell components, such as the phenolic lignin polymer of the cell wall, these bands from the C-C and C-H groups are very strong in the spectrum of cyanogenic glucosides and are likely to be observed together with the band from the nitrile moiety if dhurrin is present in the plant tissue.

**Table 1:** Assignment of the prominent bands labelled in the dhurrin spectrum (Fig. 1). δ denotes a bending vibration and ν denotes a stretching vibration. The strength of the bands is indicated by the letters: s (strong); m (medium); and, w (weak)^21^.

Wavenumber (cm^−1^)	Band Assignment (strength of band)
865	δ C-H (s)
1000	δ C-C (w)
1085	δ C-C (w)
1130	δ C-C (m)
1177	δ C-C (m)
1620	νC-C
2245	νC≡C (s)
2820	νC-H from aromatic groups (w)
2906	νC-H from aromatic groups (s)
2933	νC-H from aromatic groups (s)
2954	νC-H from aromatic groups (s)
3065	νC-H from aromatic groups (m)

### Cyanide potential (HCNp) of sorghum coleoptiles

We used bulk measurements to confirm that the *tcd1* sorghum mutant used as control was acyanogenic. Dhurrin concentration (measured as HCNp) was 5.5 mg g^−1^ of the dry mass in the coleoptiles of wild type *S. bicolor* (0.6% dry mass). This is several-fold higher than HCNp for older green seedlings reported in the literature^25,26^ but matches the 6 mg g^−1^ reported for etiolated coleoptiles^27^. The very high concentrations (35% dry mass) reported by Halkier and Møller^27^ are for the proximal end of the coleoptile, where most of the dhurrin in located, whereas here we used entire shoots. Hydrogen cyanide release was detectable from the *tcd1* mutant seedlings, albeit in very low concentrations (<0.2 mg g^−1^; Table 2); *tcd1* plants have a mutation in the CYP79A1 enzyme catalysing the first step in dhurrin synthesis, which results in loss of catalytic activity^20^. Formation of the mutated CYP79A1 protein is however not blocked, resulting in a small amount of residual activity as previously observed in this mutant^20^.

**Table 2:** Hydrogen cyanide potential (HCNp) of coleoptiles of *Sorghum bicolor* in wildtype and the *tcd1* mutant. Values are the mean of 8 replicates ±1SE.

Line	HCNp (mg g^−1^ dwt)
*S. bicolor* (WT)	5.5 ± 0.2
*tcd1*	0.18 ± 0.01

### Tissue location of dhurrin using Raman imaging

**Figure 2:**
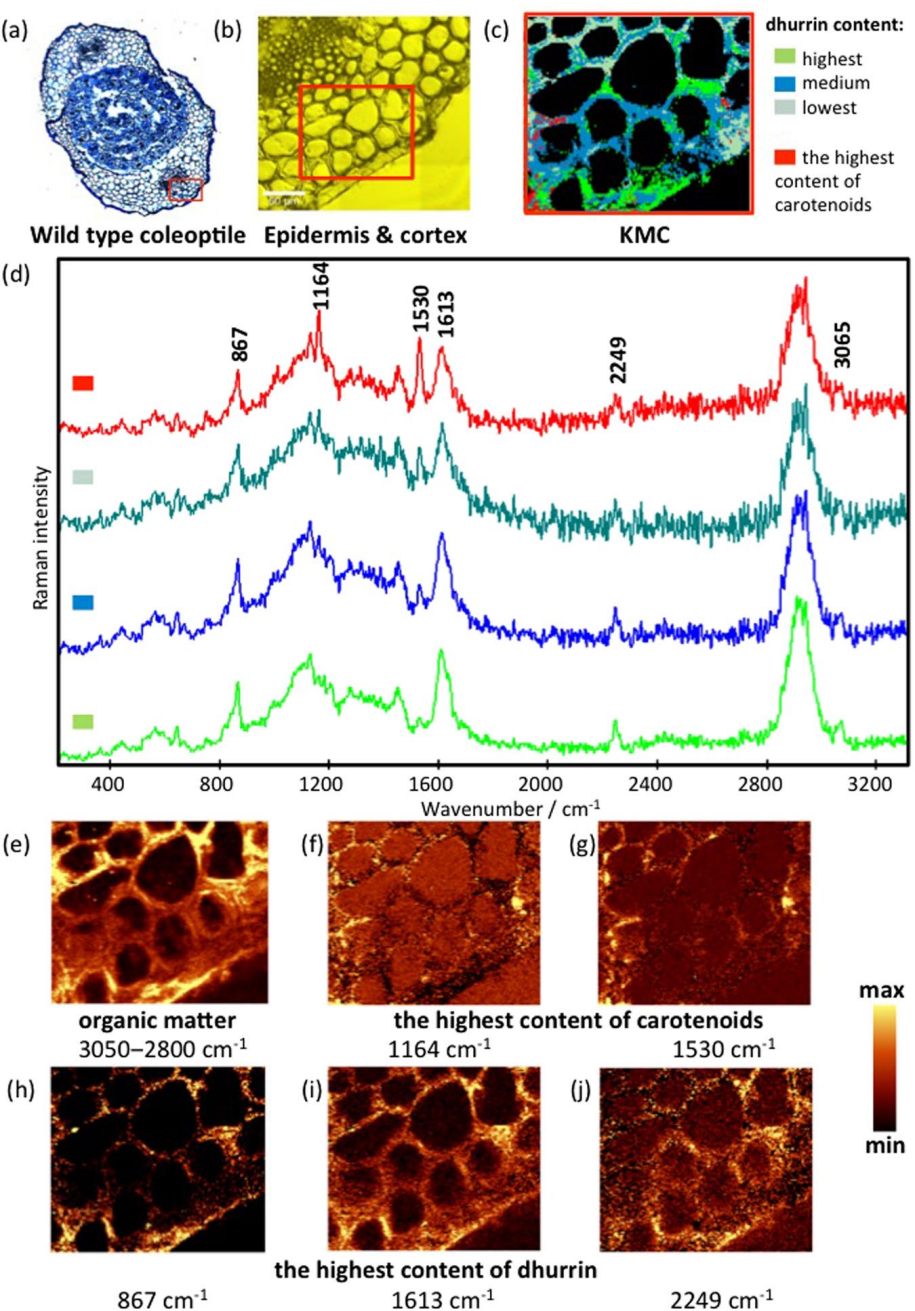
(**a**) Micrograph of toluidine blue stained cross section of the wildtype sorghum coleoptile. (**b**) Visible image from Raman microscopy of lower right-hand area of section showing epidermis and cortical cells with the investigated area of 137 × 119 μm as marked on panel a. (**c**) The KMC results, with the average Raman spectra for the main classes, including highest (green), medium (blue) and lowest (grey green) dhurrin content and the highest content of carotenoids (red). Raman intensities were normalized for C-H stretching region (2800–3015 cm^−1^) in order to compare the dhurrin content in different classes. (**d**) Assignment of bands; (**e**–**j**). Integration Raman maps obtained with a sampling density of 1 µm were made for the following. (**e**) C-H stretching band in the region of approx. 2800‒3050 cm^−1^ showing organic constituents present. (**f**,**g**) The bands at around 1164 cm^−1^ and 1530 cm^−1^, assigned to C-C and C=C vibrations of carotenoids, respectively. (**h**–**j**) The strongest bands of the dhurrin observed at 867, 1613 and 2249 cm^−1^, respectively. The yellow colour corresponds to the highest relative intensity of the integrated band. All material was prepared using cryosections.

The anatomy of the coleoptile of the germinating seedlings seen in cross-section was as previously described^28^. The developing leaves are encased in a parenchymatous cortex, surrounded by an epidermis (Fig. 2a; Supplementary Fig. S1). Two vascular strands (presumptive vascular bundles) that run along the length of the cotyledon are located in the middle of the cortex, and can be seen in cross section laterally to the central, developing primary leaf.

We employed hyperspectral imaging as a new approach to locate the cyanogenic glucoside dhurrin in the coleoptile tissue of sorghum at sub-cellular spatial resolution. Previous non-imaging approaches were dependent on the use of bulk material. In early work^23,29^, information about the cellular location of cyanogenic glucosides in sorghum was obtained by isolating protoplasts from leaves and separating epidermal and mesophyll cells using density gradient centrifugation. This work demonstrated that dhurrin was predominantly stored in the epidermal cells. Complimentary to this finding, the β-glucosidase enzyme, dhurrinase, responsible for breaking down dhurrin to release HCN, was found in these studies to be in the mesophyll^8,29,30^.

A sophisticated way to determine molecular distribution patterns from hyperspectral imaging data is to employ multivariate data analysis clustering approaches. We used K-means cluster analysis (KMC^22^), to show the location of groups of similar spectra in the Raman maps. KMC group spectra are based on spectral similarity. We found that four clusters were sufficient to define the biological molecular distribution information in the maps. Specification of additional clusters resulted in definition of the fluorescence background and of noise components in the spectra. Micrographs of the sorghum coleoptile section indicate the region of the section containing epidermal and underlying cortical cells where Raman mapping was performed (Fig. 2a,b). KMC mapping in the sorghum coleoptile, defined by the colours green, blue, grey-green and red, indicate the position of spectra from four clusters (Fig. 2c).

The average spectra from the four clusters are shown in Fig. 2d. Three of the clusters coded green, blue and grey-green indicate regions of decreasing dhurrin concentration shown by the intensity 2249 cm^−1^ band assigned to δC≡N from dhurrin. The average spectra of the four clusters are adjusted to show identical peak areas for the C-H stretching region (2800–3015 cm^−1^) to facilitate visual comparisons of the profiles. The different amplification factors used is apparent from the differences in the background noise level. As stated above, in absolute values the intensity of the 2249 cm^−1^ band thus shows significant decreases going from the green via blue to the grey-green spectrum. The green coded cluster spectra also exhibited higher intensity for the bands at 3065 and 867 cm^−1^ from C-H stretching and bending (νC-H and δC-H) modes observed in spectrum of dhurrin (Fig. 1) corroborating the view that this cluster identifies regions where dhurrin concentration is at its highest. The fourth cluster identified by KMC analysis is coded by a red colour and represents the distribution of carotenoids, with the bands at around 1164 cm^−1^ and 1530 cm^−1^, assigned to C-C and C=C vibrations of carotenoids, respectively (Fig. 2c,d).

**Figure 3:**
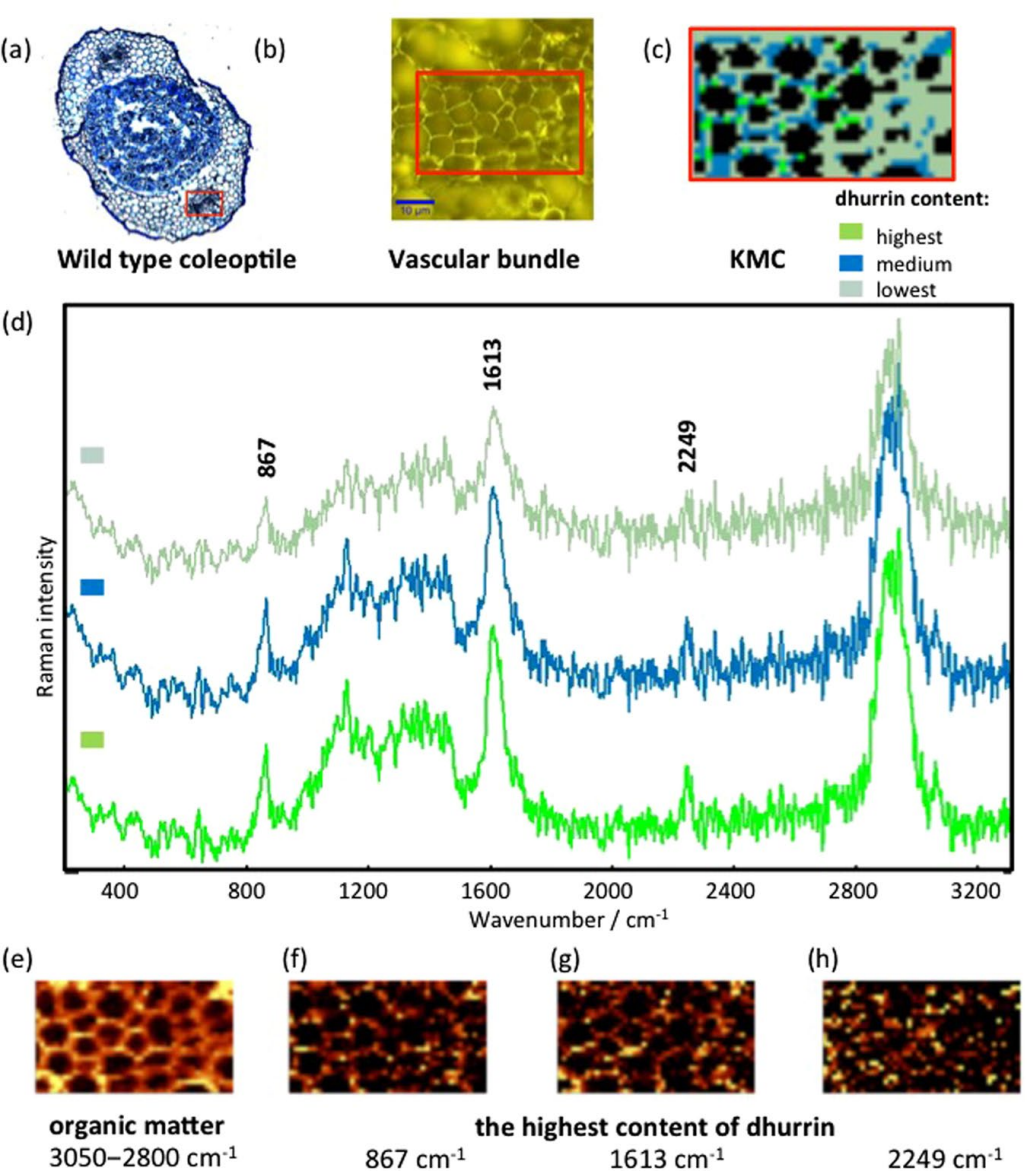
(**a**) Micrograph of toluidine blue stained cross section of the wildtype sorghum coleoptile. (**b**) Visible image from Raman microscopy of the central area of the section showing the vascular bundle region with the investigated area of 42 × 24 μm as marked on panel a. (**c**) The KMC results, with (**d**) the average Raman spectra for the main classes, including highest (green), medium (blue) and lowest (grey green) dhurrin content. Raman intensities were normalized for C-H stretching region (2800–3050 cm^−1^) in order to compare the dhurrin content in different classes. (**e**–**h**) Integration Raman maps obtained with a sampling density of 1 µm were made for the following: (**e**) C-H stretching band in the region of approx. 2800‒3050 cm^−1^ showing organic constituents present; (**f**–**h**) the strongest bands of the dhurrin observed at 867, 1613 and 2249 cm^−1^, respectively. The yellow colour corresponds to the highest relative intensity of the integrated band. All material was prepared using cryosections.

Chemical mapping (Fig. 2e–j) was carried out on the same tissue region of the sorghum coleoptile as used for cluster mapping (Fig. 2c). The Raman mapping intensity of the bands from C-H stretching vibrations in the spectral region from 3050 to 2800 cm^−1^ is related to the concentration of all organic constituents present in the sample (Fig. 2e), and demonstrates that the highest concentration of constituents is found at the periphery of the cells. This correlates with the position of the cell walls in the visible image (Fig. 2a). In the visible images, the cytoplasm was observed adpressed towards the plasmamembrane and thus in close proximity to the cell wall (see Supplementary Fig. S1), implying that scattering from constituents in the cytoplasm may have contributed to the image (Fig. 2e). The lack of scattering from the interior regions of most cells in this map is consistent with the presence of a large central vacuole. Surprisingly no dhurrin signal was observed from the vacuole which in previous studies had been pointed out as the main storage site for dhurrin in sorghum^23,29^. The distribution of carotenoids is revealed by the intensity of the two strong bands at 1164 cm^−1^ and 1530 cm^−1^ (Fig. 3f,g). Hotspots in the distribution correlate well with the pattern shown by the red cluster in Fig. 2c. Figure 2h–j show the intensity of strong bands consistent with the spectrum of dhurrin (Fig. 1). The map of intensity at 2245 cm^−1^ (Fig. 2j) indicates best the distribution of dhurrin, as this band is unique to dhurrin whereas the band at 867 (Fig. 2h) may also be assigned to cell wall components such as lignin. Nevertheless, all three maps show a similar intensity distribution to the green cluster (Fig. 2c) with obvious “hotspots” in many locations with greatest intensity surrounding cortical cells and less around epidermal cells and in the cuticle. The “hotspots” identified with dhurrin in Fig. 2j also corresponded to the highest intensity area in the map for organic constituents present (Fig. 2e) presumably due to the very intense band from dhurrin in the spectral range 3050–2800 cm^−1^ (Fig. 1) and the high concentrations found in these regions. Indeed, the existence of highly concentrated regions containing dhurrin strongly suggests that these represent original tissue locations in the intact tissues and were not the result, for example, of dhurrin being redistributed during histological sectioning. A very similar distribution for dhurrin shown in Fig. 2c and j was observed in all wildtype coleoptile tissues that were examined (Data available on request).

Cluster mapping also identified regions of high dhurrin concentration in the developing vascular strands of the sorghum coleoptile shown by the intensity hotspots for the green cluster (Fig. 3c). This pattern was observed in all seedlings examined. The location of these intensity hotspots was correlated with intensity hotspots for the bands at 2245, and 867 cm^−1^ assigned to dhurrin in the chemical maps (Fig. 3h,j). Spectra from the vascular tissue exhibited a poorer signal to noise ratio than those from the epidermis and underlying cortical tissue, presumably due to greater light scattering. This was overcome to some extent by using increased laser power in these measurements, but restricted by the need to avoid burning of the sample. Interestingly, the regions of high concentration were predominantly in the interior part of the vascular bundle, with respect to the coleoptile, corresponding with the location of the presumptive phloem (Supplementary Fig. S1).

### Absence of dhurrin in *tcd1* mutant plants confirmed by Raman imaging

**Figure 4:**
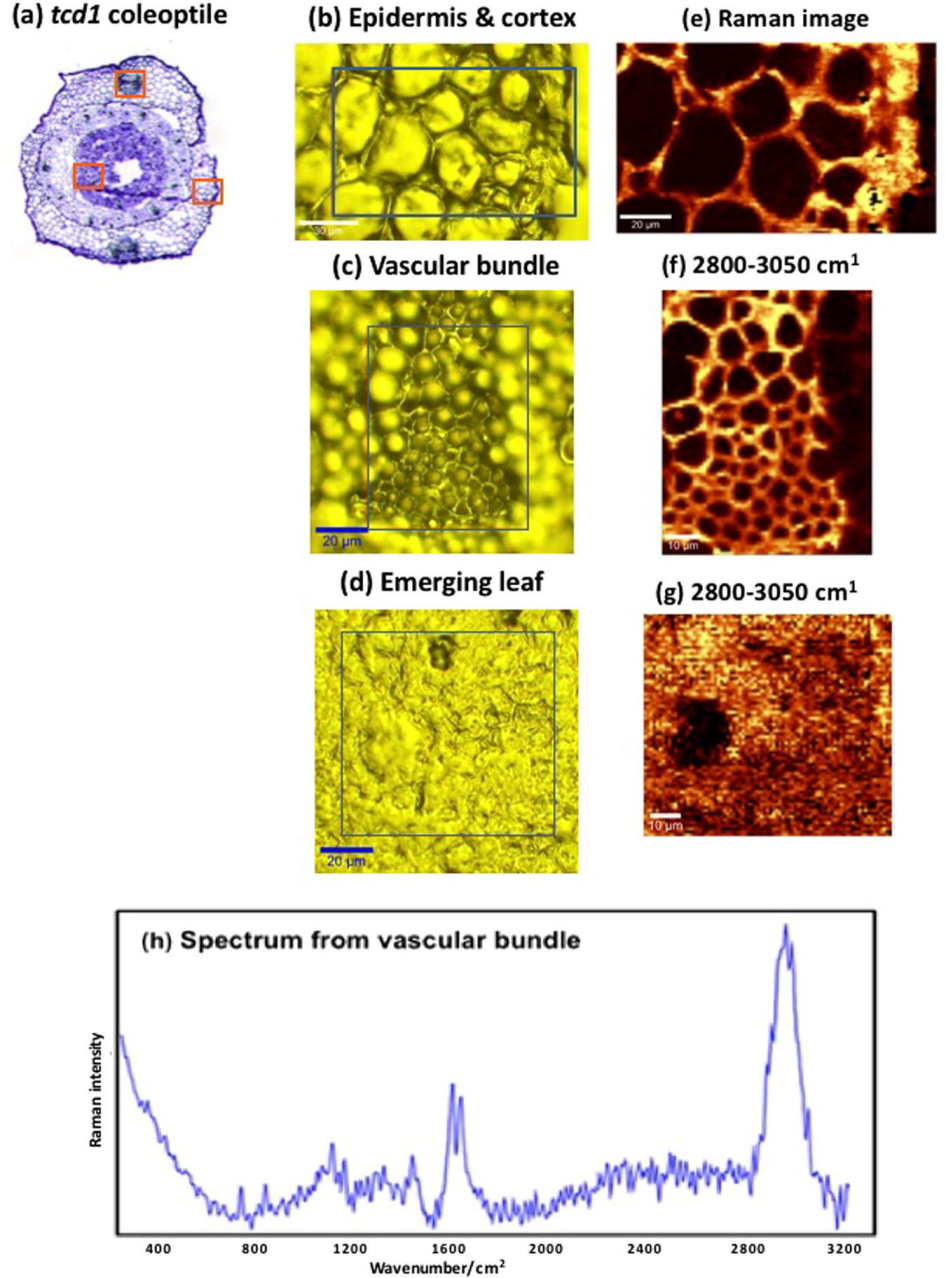
(**a**) Toluidine blue stained image of the *tcd1* coleoptile. The Raman-scanned areas are marked by red boxes. Visible images of (**b**) the epidermis and cortex; (**c**) vascular bundle; (**d**) emerging leaf; Corresponding Raman images (at 2800–3050 cm^−1^) of the three tissue types in the *tcd1* coleoptile (**e**–**g**). The 2245 cm^−1^ nitrile (ν C≡N) band is absent in all tissues, indicating absence of dhurrin. (**h**) Average spectrum of 50 spectra randomly selected from the vascular bundle map. All material was prepared using cryosections.

In contrast to the observations in the wildtype sorghum coleoptiles, no spectra that showed the characteristic band at 2245 cm^−1^ from dhurrin, or any other band characteristic of dhurrin, were detected in tissues from the *tcd1* mutant. This is shown by an average spectrum obtained from 50 randomly selected spectra from the vascular bundle in a *tcd1* mutant coleoptile (Fig. 4). The same profile was obtained for all *tcd1* coleoptile sections examined. The main observed spectral bands were the doublet at 1600 and 1630 cm^−1^ from lignin and C-H stretching bands centred around 2900 cm^−1^ from the total organic constituents present in the tissue^31,32^. There was no evidence of any band at 2245 cm^−1^ nor the band near 856 cm^−1^ that is characteristic of dhurrin. KMC analysis showed the same result (data not shown). Similarly, spectra from maps from other tissue regions in the coleoptile of the *tcd1* mutant showed no evidence of dhurrin bands (data not shown). These results are consistent with the results of the bulk tissue analysis (Table 2) showing that the tissue from the *tcd1* mutant plant contains very little dhurrin. Importantly, these trace amounts of dhurrin are evidently below the limits of detection by Raman spectroscopy in the tissues examined.

### Biological implications of dhurrin distribution observed in wildtype sorghum coleoptiles

Chemicals that provide defence against herbivores are typically stored in locations where they provide the most benefit to the plant^4^. The presence of dhurrin in the outer layer of the cotyledon – the epidermis and underlying cortex – provides a first line of chemical defence against attack. Localisation of cyanogenic glucosides to external tissues is also seen in cassava^33^ and for glycoalkaloids in potatoes^34^. Studies using protoplast isolation also found that dhurrin was located in peripheral epidermal cells, but not the outer cortical parenchyma^8,23,30^. Kojima, *et al*.^8^ notes specifically that very little dhurrin was found associated with bundle sheath cells in their study. The plants used in the current study were much younger which may explain this difference^35^. In the pre-emergent stage, young sorghum leaves are different biochemically and anatomically displaying characteristics of C3 photosynthesis rather than the C4 normally found in sorghum^33^. There were no obvious bundle sheath cells, characteristic of C4 plants, in the sections described here.

The highest concentrations of dhurrin are found at the very tip of the cotyledons^27^. Not only would this protect the growing tip from herbivores, it may also be the primary site of synthesis for the developing shoot. In cassava, the cyanogenic glucosides are almost exclusively synthesised in the growing tips and then transported to other parts of the plant, including the roots, via the phloem^36^. It is tempting to attribute the location of dhurrin within the vascular tissue to the phloem, as cyanogenic glucosides have previously been found to be associated with phloem^37^ and have been reported to be transported in the phloem as diglucosides in several species^5^ including sorghum^38^. In the current study, the lack of phloem-specific staining in the young cotyledons provides no evidence for the presence of a functional phloem (See Fig. 3a). It may be that the dhurrin is associated with the vascular cambium and presumptive xylem. The vascular cambium is an area of very active growth and it could be that the dhurrin is delivering reduced nitrogen for protein synthesis at this site as known for other actively growing tissues in sorghum^39,40^. In black cherries, catabolism of amygdalin during seed germination provides reduced nitrogen and carbon for early seedling development^36^.

The observed alignment of dhurrin in the adpressed cytoplasm and cell walls was unexpected. Cyanogenic glucosides have previously been reported present in woody tissue such as the pericarp of developing fruits in *Macadamia*^41^ and black cherry^36^. This would not have been observed in studies that used isolated protoplasts, as the cell walls are digested away as part of the isolation process.

We explored whether the presence of dhurrin in the cytoplasm could have arisen from leakage from disrupted cells by testing for cell viability using Hoechst and TO-Pro stains. These tests confirmed that the cells were intact and that the membranes retained their integrity (Supplementary Fig. S1). Separation of dhurrin from the specific glucosidase is necessary for the plant to avoid autotoxity. This does not preclude a cytoplasmic location of dhurrin, but it would likely require either a tissue level based separation as reported in sorghum and almonds^42^. In the present study, we were unable to resolve whether the dhurrin localized in the cytosol was contained in any kind of separate compartment. In cassava, cyanogenic glucosides in the petioles and stems are stored in vesicles in the latex, which effectively isolates them from the high concentration of β-glucosidase and hydroxynitrile lyase in the latex itself^43^. The recent discovery of membrane-less organelles^44,45^ opens up a whole range of new possibilities for novel mechanisms for subcellular compartmentalization.

## Conclusion

Raman spectroscopy was used to identify the *in situ* subcellular localization of dhurrin in sorghum in young seedlings. In the two different tissue types analysed, dhurrin was localized within the cytoplasm, although at this scale it was not possible to see if it is localized to particular organelles or vesicles. This type of spectroscopic analysis requiring no preliminary chemical treatment of the plant material has the potential to transform the understanding of the spatial localization of cyanogenic glucosides and other specialized metabolites at the subcellular level in plant tissue.

## Methods

### Plant material

*Sorghum bicolor* (L.) Moench seeds of two lines, an inbred parent line (Pacific Seeds, Toowoomba Qld., Australia) and the EMS-generated *totally cyanide deficient 1* (*tcd1*) mutant^20^ were placed on gauze covered screens in water^27^ and germinated in the dark at 22.5 °C for 4–5 days. Coleoptiles were harvested and processed directly for sectioning and cyanide assays.

Coleoptiles were frozen at −20 °C in 10 × 10 × 5 mm cryomolds containing the inert support medium Tissue-Tek® O.C.T. following Fischer *et al*.^46^. The temperature was lowered to −50 °C by immersing the molds in isopentane (SIGMA-ALDRICH, Australia, Catalogue #270342,), which had been pre-cooled in liquid nitrogen. The samples were kept at ~50–55 °C for 1 min, then placed on dry ice or stored at −80 °C. Transverse sections (10 µm) were cut on a Leica CM1850 cryostat and placed on poly-L lysine coated slides (Plysine®, Thermo Scientific, Australia). Serial sections were used for either Raman imaging or histological analysis.

### Raman microspectroscopic mapping

Raman imaging of sorghum tissue sections was performed on a WITec confocal CRM alpha 300 Raman microscope (WITec Wissenschaftliche Instrumente und Technologie GmbH, Ulm, Germany). At least six tissue sections from coleoptiles of both wild type and *tcd1* mutant plants were examined. The spectrometer was equipped with an air–cooled solid state laser operating at 532 nm and a CCD detector, which was cooled to −65 °C. The laser was coupled to a microscope via a single mode optical fibre with a diameter of 50 µm. The scattered radiation was focused onto a multi–mode fibre (50 μm diameter) and a monochromator. The integration time for a single spectrum was 2 s with a spectral resolution equal to 3 cm^−1^ and a mapping step distance of 0.5 µm. The laser intensity in the focus spot was <5 mW in all measurements. Raman is a transmission spectrum and therefore each measurement interrogates the entire depth of the section at each position. The monochromator of the spectrometer was calibrated using the Raman scattering line produced by a silicon plate (520.7 cm^−1^). For Raman map collection a Nikon (x60/1NA) objective was used. Spectra were acquired from pure dhurrin^24^ by placing a few milligrams onto a gold coated glass slide and then using 0.1 mW of laser power with 532 nm excitation. Using a 532 nm excitation wavelength of the laser and the numerical aperture of the microscope objective of 1NA, the maximal possible spatial resolution is equal to 0.33 µm, however this was limited in these experiments to 0.5 µm by the mapping density.

### Image analysis

Raman data analysis was performed with Opus^TM^ and WITecsoftware^TM^. Raman maps were generated based on the integration of marker bands and were obtained without pre–processing. Cluster analysis was carried out after cosmic ray spike removal and background subtraction. K–Means Clustering (KMC) results were obtained with the Manhattan distance algorithm^47^ and are complementary to the analysis based on the integration of the specific marker bands.

### Histological methods

Sections adjacent to the ones prepared for Raman imaging were stained with an aqueous solution containing 0.05% toluidine blue O in 0.1 M phosphate buffer at pH 6.8^48^. In order to confirm the integrity of the cells, additional representative sections were stained with Hoechst (#33342, Thermo-Fisher Scientific,) and TO-PRO (#T3605, Thermo-Fisher Scientific)^49^. Hoechst 33302 is a membrane permeable, nuclear stain and can be used to test nuclear damage and cell viability^50^. TO-PRO-3, another fluorescent stain specific for nuclei, is impermeable to live cells but penetrates compromised membranes characteristic of dead cells and served as an alternative test of cell viability and integrity.

### Hydrogen cyanide assays

Dhurrin concentration was measured as the hydrogen cyanide potential (HCNp) on shoots of 3-day-old etiolated seedlings^20^. Eight replicates of wild type and *tcd1* plants were analysed by placing a single whole coleoptile (cut into 3 mm segments) into a vial and adding 250 µL β-glucosidase (β-D-Glucoside glucohydrolase, Sigma, EC 3.2.1.21 in 0.1 M citrate buffer pH 5.5) to ensure complete hydrolysis of dhurrin. Tissue was disrupted by repeated freeze-thaw cycles. Evolved HCN was captured in 200 µL of 1 M NaOH solution and measured as NaCN in a colorimetric assay and the HCNp of the sample determined as mg g^−1^ dry weight of tissue analysed^51^.

## Electronic supplementary material

Supplementary Figure
