# Supplementary material for: Label-free Raman hyperspectral imaging analysis localizes the cyanogenic glucoside dhurrin to the cytoplasm in sorghum cells
Source: Sci Rep. 2018 Feb 9;8:2691. doi: 10.1038/s41598-018-20928-7 (PMC5807435; doi:10.1038/s41598-018-20928-7)
Supplement: Supplementary file 1 — Supplementary Figure [file 41598_2018_20928_MOESM1_ESM.pdf]

Label-free Raman hyperspectral imaging analysis localizes the cyanogenic glucoside dhurrin to the cytoplasm in sorghum cells

Philip Heraud[1,2], Max F. Cowan[3], Katarzyna Maria Marzec[4,5], Birger Lindberg Møller[6,7], Cecilia K. Blomstedt[3], Ros Gleadow[3*].

[1]*Department of Microbiology, Faculty of Medicine, Nursing and Health Sciences, Monash University, Wellington Rd, Clayton, Vic., 3800 Australia.*

[2]*Centre for Biospectroscopy, School of Chemistry, Monash University, Wellington Rd, Clayton, Vic., 3800 Australia.*

[3]*School of Biological Sciences, Faculty of Science, Monash University, Wellington Rd, Clayton, Vic., 3800 Australia.*

[4]*Jagiellonian Centre for Experimental Therapeutics (JCET), Jagiellonian University, Bobrzynskiego 14, Krakow, Poland.*

[5]*Center for Medical Genomics (OMICRON), Jagiellonian University, Kopernika 7C, 31–034 Krakow, Poland.*

[6]*Centre for Synthetic Biology, University of Copenhagen, Thorvaldsensvej 40, 1871 Frederiksberg C, Denmark.*

[7]*VILLUM Center for Plant Plasticity, University of Copenhagen, Thorvaldsensvej 40, 1871 Frederiksberg C, Denmark.*

***Corresponding author:**

Prof. Ros Gleadow

School of Biological Sciences, Monash University, Wellington Rd, Clayton, Vic., 3800 Australia.

Email: ros.gleadow@monash.edu; Phone: +61 3 99051667; Fax: +61 3 99055613.

Supplementary Information

Supplementary Figure 1

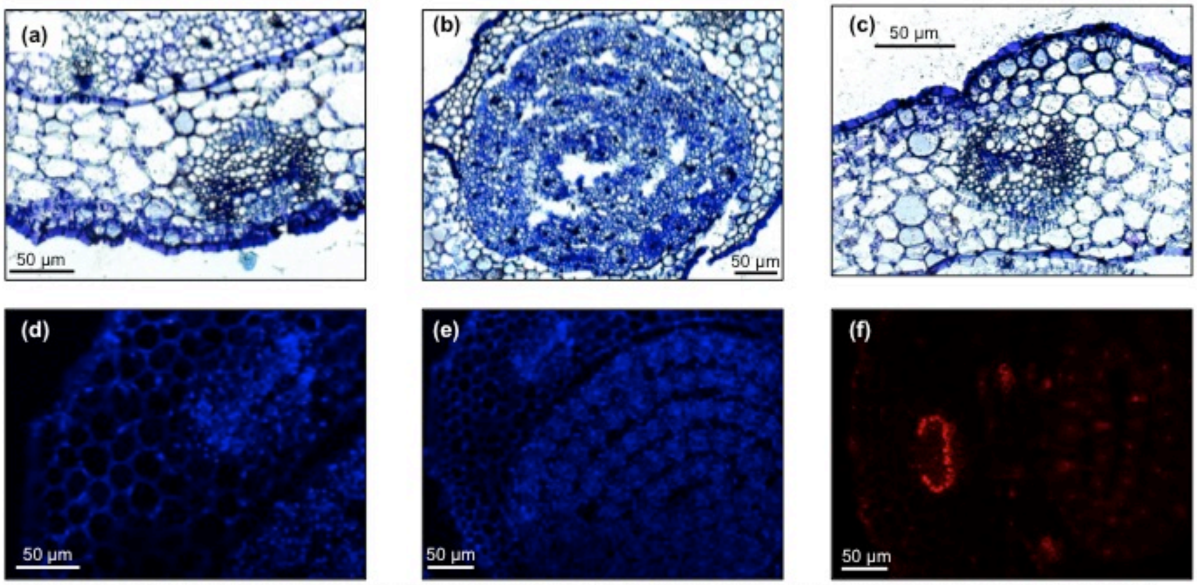

Supplementary Figure 1. Transverse cryosections of etiolated sorghum coleoptiles stained with Toluidine Blue (a-c), Hoeschst 33302 (d-e) and TO-PRO (f) showing details of the cortex (a, d), the pre-emergent true leaf; (b, e) and the vascular bundle (c, f). Toluidine Blue can be used as a diagnostic tool on fresh and frozen sections as follows: trachery elements stain green- or blue-ish; lignin stains green-blue; the sieve tubes of the phloem appear red; collenchyma and parenchyma are red-purple; cellulose is blue; callose and starch remain unstained (O'Brien *et al.*, 1964). In these sections the cell walls of prospective vascular tissue are purple, and thus do not appear to be lignified, nor does there appear to be mature phloem. Note the lack of bundle sheath cells around the vascular traces. Hoeschst is a membrane permeable, nuclear stain and can be used to test nuclear damage and cell viability (Chazotte, 2011). TO-PRO-3, another fluorescent stain specific for nuclei, is impermeant to live cells but penetrates compromised membranes characteristic of dead cells and served as an alternative test of cell viability and integrity. Both nuclear stains confirm that cells retain their integrity with this method of sectioning.
